# Survivorship of Reverse Shoulder Arthroplasty According to Indication, Age and Gender

**DOI:** 10.3390/jcm11102677

**Published:** 2022-05-10

**Authors:** Mikaël Chelli, Pascal Boileau, Peter Domos, Philippe Clavert, Julien Berhouet, Philippe Collin, Gilles Walch, Luc Favard

**Affiliations:** 1 University Institute of Locomotion and Sports, University Hospital of Nice, 06000 Nice, France; mikael.chelli@gmail.com (M.C.); boileau.p@chu-nice.fr (P.B.); 2 Barnet and Chase Farm Hospitals, Royal Free London NHS Foundation Trust, Barnet EN5 3DJ, UK; peter.domos@nhs.net; 3 University Hospital of Strasbourg, 67000 Strasbourg, France; philippe.clavert@medecine.u-strasbg.fr; 4 University Hospital of Tours, 37000 Tours, France; julien.berhouet@univ-tours.fr; 5 West Locomotion Institute, 35760 Saint-Grégoire, France; collin.ph@wanadoo.fr; 6 Santy Orthopedic Center, 69008 Lyon, France; gilleswalch15@gmail.com

**Keywords:** reverse total shoulder arthroplasty, outcomes, survivorship

## Abstract

Background. The indications for reverse shoulder arthroplasty (RSA) have been widely expanded, but only a few studies report the long-term survival of these implants. Our objective was to report the long-term survivorship of a large series of RSAs implanted for different etiologies. Methods. A retrospective multicenter study including all the RSAs was performed in six shoulder-specialized centers with at least 2 years of follow up. We reviewed 1611 RSAs, operated between 1993 and 2010, including 497 cuff-tear arthropathies (CTA), 239 revision RSAs, 188 massive cuff tears (MCT), 185 fracture sequelae (FS), 183 failed previous cuff repairs (FCR), and 142 primary osteoarthritis (POA). The mean follow-up was 5.6 ± 3.9 years (range 2–20). Results. Overall, 266 RSAs (16.5%) had at least one complication leading to 64 reoperations (4.0%) and 110 revision surgeries (6.8%). The most frequent complications were infection (3.8%), instability (2.8%), and humerus-related complications (2.8%). At 10 years, the survival without revision surgery was 91.0% in primary RSAs and 80.9% in revision RSAs for failed arthroplasty (*p* < 0.001). In the primary RSA group, MCT and FCR led to 10-year survivals for over 95% but fracture sequelae and tumors had the lowest 10-year survivals (83.9% and 53.1%). Younger patients had a lower 10-year survival. In revision RSAs, male patients had a significantly lower survival than females (72.3% vs. 84.5% at 10 years, *p* = 0.020). Discussion. Primary RSA for cuff-deficient shoulders or POA leads to a high 10-year survival, but revision RSA or primary RSA for FS and tumors are at high-risk for revision. Surgeons should be aware of high rates of complications and lower survival rates of RSA in younger patients, in males, and in RSAs for revision surgery.

## 1. Introduction

The currently used type of reverse shoulder arthroplasty (RSA) was designed in 1985 by Paul Grammont [1,2] and approved in the United States in 2003. It was initially aimed at patients with cuff tear arthropathy [3,4], but indications have expanded over the years to the revision of failed arthroplasties and several other pathologies [5,6,7]. Mid-term retrospective studies and the joint registries (available in Australia, Denmark, Italy, Norway, Sweden, and United Kingdom [8,9,10,11,12]) have confirmed overall survival rates of over 90% for RSAs in the short-term or mid-term. However, these registries also combine a large number of different implants, surgeons, and operative techniques. Multicenter retrospective studies may overcome some of these biases by merging a large number of patients operated by a limited number of surgeons with similar operative techniques and rehabilitation protocols, while also allowing for a more detailed analysis of clinical notes and relevant pre- and postoperative imaging. These techniques can provide some important details for what patients and surgeons could expect from RSAs.

Our group of surgeons had experience with RSAs for more than 20 years, and our objective was to report this experience to assess the long-term survival of RSAs in a large series of patients with different etiologies operated in shoulder-specialized centers.

Our hypothesis was that the long-term survival of an RSA would depend on the indication for an RSA, the gender, and the patient’s age at the time of surgery.

## 2. Materials and Methods

### 2.1. Study Design

We conducted a retrospective study including all the patients who were operated on with a Grammont-style RSA between 1993 and 2010 in six shoulder-specialized centers. Complications, reoperations, and revisions were extracted from the medical records. The indications for RSAs were consistent between centers: cuff-tear arthropathy (Hamada IV and V), massive and irreparable rotator cuff tear with pseudoparalysis or previous failed cuff repair, primary osteoarthritis with B2/C glenoid and/or associated cuff tear, and malignant tumors.

Patients were followed clinically and radiologically on a regular basis by surgeons. Complications, revisions, and standard x-rays were retrieved from medical records and assessed by two specialized shoulder surgeons (LF and MC).

A revision surgery was defined as any surgical procedure following the index surgery for which a glenoid and/or humeral implant or part of the implant was changed, added, or removed.

A reoperation was defined as any surgical procedure following the index surgery for which all the implants were retained, such as a reduction in dislocations, a washout and debridement, or a hematoma evacuation.

### 2.2. Patient Demographics

Between 1993 and 2010, 1611 RSAs were implanted in 1462 patients, at a mean age of 73.2 years (±9.0, range 18–94), including 1088 female (74.4%) and 374 male patients. In 149 cases (10.2%), patients had bilateral RSAs. Patients were followed for a mean of 5.6 years (±3.9, range 2–20). The distribution was as follows:

- 0 to 2 years: 354 shoulders;
- 2 to 5 years: 348 shoulders;
- 5 to 10 years: 661 shoulders;
- 10 to 15 years: 225 shoulders;
- 15+ years: 23 shoulders.

The cuff-deficient shoulders (Massive Cuff Tear + Cuff Tear Arthropathy + Failed cuff repairs) represented half of the indications of RSA (Table 1).

### 2.3. Surgical Technique

All the patients were operated on in the beach-chair position with an appropriate prophylactic antibiotic cover. The six surgeons used similar operative techniques and rehabilitation protocols. A deltopectoral approach was used in 89% of the cases, and an anterosuperior approach was used in 11%. A medialized (Grammont type) Aequalis Reverse (Wright–Tornier, Memphis, TN, USA) RSA was used in 79.6% of cases, and the Delta III (Depuy, Warsaw, IN, USA) was used in 20.4% of cases. The humeral stem was cemented in 88%, and the glenosphere was sized at 36 mm in 86% of cases and at 42 mm in 14% of cases and lateralized with a BIO-RSA in 21% of cases. A tendon transfer to restore active external rotation (L’Episcopo: Latissimus Dorsi ± Teres Major) was performed in 47 cases (3%).

### 2.4. Statistical Analysis

Numeric outcomes were expressed by their mean (±standard deviation), and discrete outcomes were expressed by their absolute and relative frequencies (%). The Fisher’s exact test was used for between-group comparisons. We used the Kaplan–Meier method to estimate the survival probabilities and their pointwise 95% confidence intervals. The log-rank non-parametric test was used to compare the survival distributions. The significance was set at *p* < 0.05. Statistical analysis was performed with EasyMedStat (www.easymedstat.com; version 2.0, France) (accessed on 12 March 2018).

## 3. Results

### 3.1. Postoperative Complications

The most frequent complications were instability (n = 61, 3.8%) and infection (n = 45, 2.8%) (Table 2). The most frequent causes for revision were infection (n = 37, 2.3%), instability (n = 24, 1.5%), glenoid loosening (n = 22, 1.4%), and humeral loosening (n = 14, 0.9%).

### 3.2. Survival Analysis

Overall, 266 RSAs (16.5%) had at least one complication leading to 64 reoperations (4.0%) and 110 revision surgeries (6.8%). At 10 years, the survival without revision was 91.0% (87.9–93.3%) in primary RSAs and 80.9% (73.6–86.3%) in revision RSAs for failed arthroplasties (Table 3).

**Figure 1:**
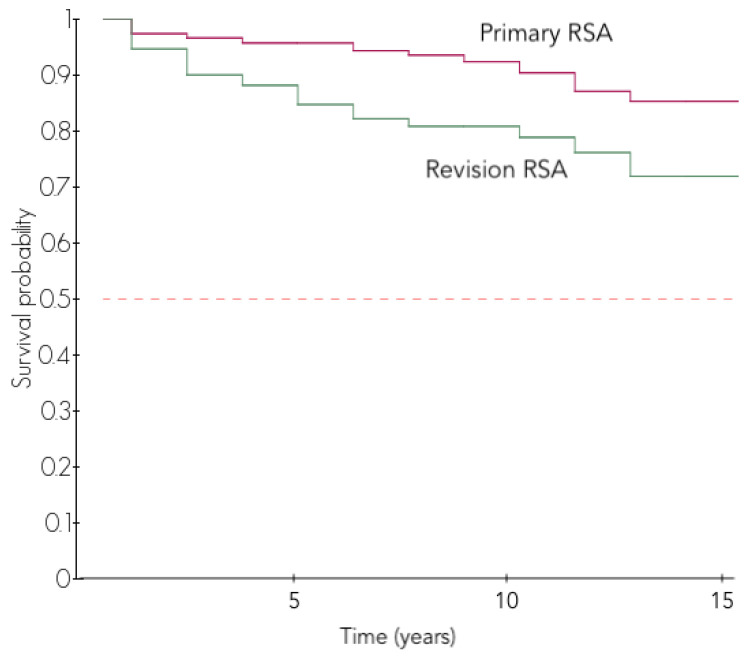
Revision-free survival in primary RSA and revision RSA.

The reoperation-free survival and the revision-free survivals were both higher in primary RSAs as compared to revision RSAs (*p* = 0.002 and *p* < 0.001, Figure 1). No difference was observed between the revisions of HA and the revisions of other arthroplasties (*p* = 0.441).

### 3.3. Predictive Factors for Survival without Revision Surgery

#### 3.3.1. Influence of Gender and Age

**Figure 2:**
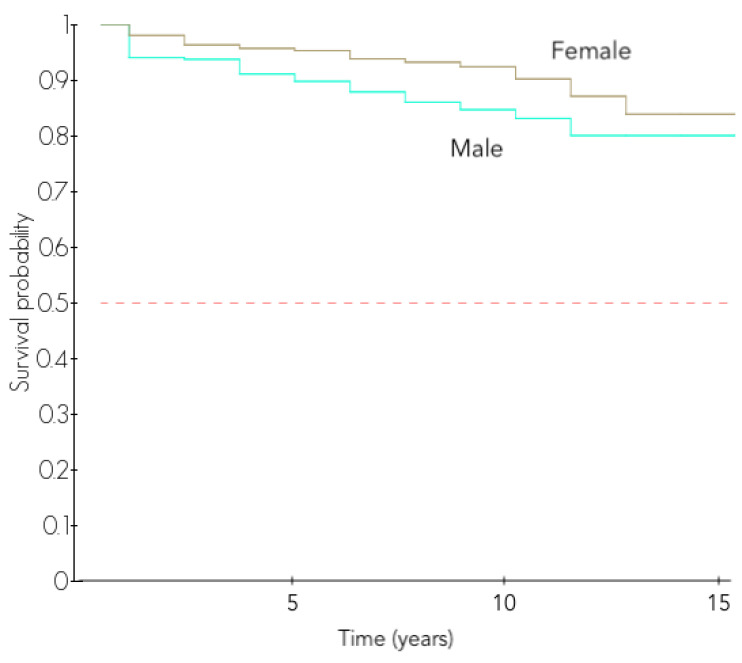
Revision-free survival according to the gender.

The rates of complications and revision surgeries were higher in male patients: the 5-year and 10-year revision-free survivals were 90.3% and 83.2% in male patients and 95.4% and 91.5% in female patients (*p* < 0.001, Table 4 and Table 5, Figure 2).

**Figure 3:**
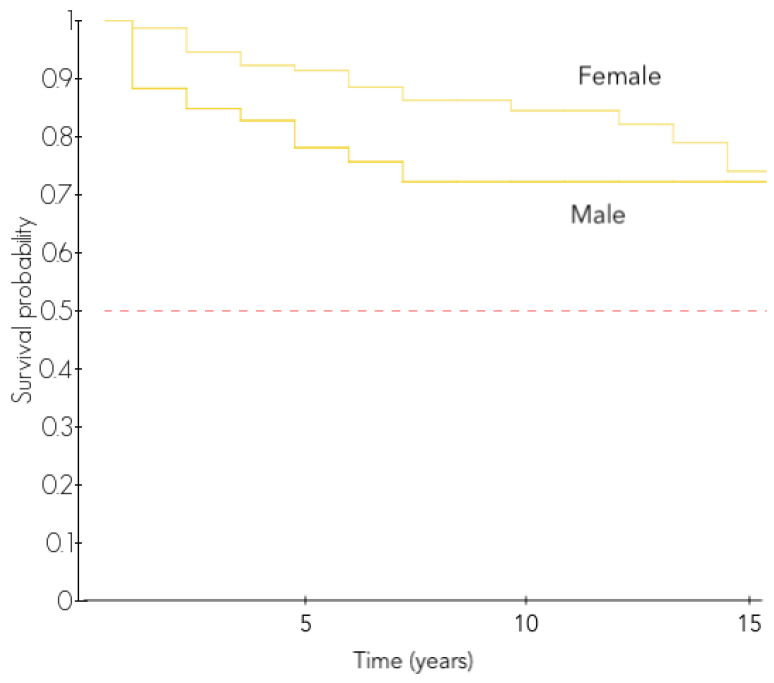
Revision-free survival of 239 revision RSA according to gender.

However, after stratification over the etiology, this difference was found only in the revision RSA group (72.3% vs. 84.5% at 10 years, *p* = 0.020, Figure 3), and was not found in any other etiology.

**Figure 4:**
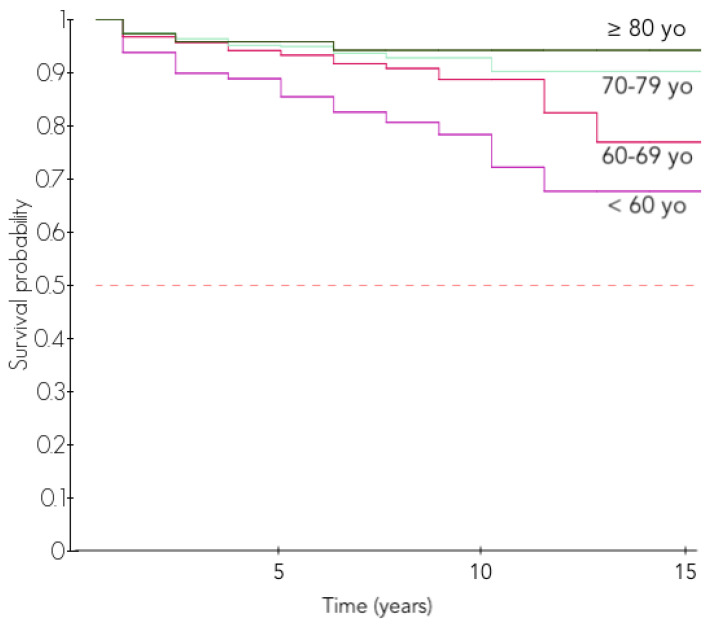
Revision-free survival according to the age at surgery.

A younger age at surgery was associated with higher rates of complications and revisions: patients younger than 60 years at the time of RSA had a 10-year survival of 75.7% while patients between 60 and 69, 70 and 79 and over 80 years had a 10-year survival of 88.8%, 91.3% and 94.3% respectively (*p* < 0.001, Table 4, Figure 4).

This association was maintained after stratification over gender with males and females requiring more revision surgeries when operated younger (Table 5). In the 70–79 age group, the males had more revisions than the females (8.5% vs. 4.5%; *p* = 0.034) but the difference was not significant for other age groups. Revision RSA performed in male younger than 60 years led to a 10-year survival rate of 72.3%.

The indication for RSA was also associated with age (*p* < 0.001), with the younger patients being operated on for tumors (51.9 years), instability arthropathy (68.9 years), and revision (69.0 years) and with the older patients being operated on for acute fractures (80.1 years), CTA (76.3 years), POA (76.1 years), and MCT (73.3 years).

#### 3.3.2. Influence of Diagnosis

The rates of complication and revision surgery varied according to the etiology (*p* < 0.001). The highest revision rates were observed in tumors (20%), revision RSAs (16%), and fracture sequelae (11%); the lowest rates were observed in instability arthropathy (0%), rheumatoid arthritis (2%), and acute fractures (2%) (Table 6).

## 4. Discussion

In this series, primary RSA led to a 10-year revision-free survival rate of over 90% and a 15-year survival rate of 85%. This 10-year rate is comparable to those reported in retrospective studies [13,14,15,16,17,18] and national joint registries [8,12,19]. For rotator cuff arthropathy, the UK registry reports a 10-year revision rate of 5.9% and the Australian registry reports a 7-year revision rate of 5.6%. The retrospective series reports 10-year overall survival rates between 88% and 93% for different etiologies (Table 7).

The most frequent complications leading to revision surgery were infection, instability, and loosening, which is consistent with the systematic review of 21 studies and 782 RSAs by Zumstein et al. [20]. These complications differ from what can be found with an anatomical total shoulder arthroplasty, where the most frequent complications are related to the glenoid and the cuff [21,22]. Contrarily, in an RSA, the humerus is the main source of complications with 2.8% of patients suffering a humeral loosening or a humeral fracture, while glenoid loosening was found in only 1.6% of cases [23].

Revision RSA was associated with a lower survival at every time point compared to primary RSA (80.4% vs. 91.0% at 10 years, *p* = 0.002), as reported by other authors in previous studies [6,24,25]. Saltzman et al. [25] found that the revision status was the most significant predictor of intraoperative and postoperative complications in a retrospective series of 137 patients. Zumstein et al. [20] reported a complication rate that was more than twice as high in cases of revision compared to primary RSAs (33% vs. 13%). Although revision RSA is sometimes the only solution after a failed unconstrained arthroplasty, surgeons should be aware that it is a challenging surgery with high rates of complications and revision surgeries. The main complication in a revision RSA is a low-grade infection.

The diagnosis for a primary RSA is one of the main predictive factors of postoperative complications and revision surgeries. While MCT and failed cuff repairs led to 10-year survivals of over 95%, fracture sequelae and tumors had much lower survivals (83.9% and 53.1% at 10 years, respectively). The latter two etiologies and revision RSAs are associated with major complications such as instability and humeral loosening [26,27,28,29] because of the frequency of missing proximal bone stock preventing rotational control of the stem [30]. The 10-year survival of an RSA for primary osteoarthritis was high (90.3%), which confirms it is a viable option in cases of severe humeral subluxation and/or subluxation [31,32,33]. RSA for rheumatoid arthritis led to a 10-year survival of 97.6%, which is superior to that reported for HA and TSA in the same indication [34,35,36].

We observed that younger patients were more likely to have revision surgery after an RSA. The 10-year survival of patients younger than 60 at the time of surgery was only 75.7% vs. a 94.3% survival for patients older than 80 years at the time of the RSA. This might be related to the higher activity in younger patients and less favorable indications such as tumors and revision RSAs. The influence of age could not be highlighted in previous studies comparing younger and older patients, potentially due to the lack of statistical power and the shorter follow-up time [37,38,39].

Male patients had a higher risk of revision as it has been found in the Nordic registry [8]. However, the Australian registry found this difference only in the first three months after surgery [19]. After stratification over the indication, we found a higher risk for males that were only in the revision RSA group. These results may show that this higher revision rate of male patients in the revision RSA group and in the first three months is due to a higher risk of infection, as demonstrated by the Norwegian registry [40] and the Mayo clinic study [41].

Our study is limited by its retrospective design. Subjective and functional outcomes were not assessed and remain important factors for surgeons to decide which surgical option they should consider. However, this study is unique as it reports a 17-year experience of a group of shoulder-specialist surgeons with two models of Grammont-type RSAs. To the best of our knowledge, this series represents the largest series of RSAs published in the literature, with the longest follow-up (up to 20 years). The large number of patients reviewed and the long-term follow up allowed for the analysis of survival rates and complications in RSAs implanted for a wide range of conditions. This source of information is of paramount importance for surgeons as it also provides further useful information to inform the patients about the expected survivorship of these implants according to their etiology and age.

**Table 7:** Mid-term and long-term survivals of reverse shoulder arthroplasty reported in the literature.

First Author	N =	Indication	FU (y)	Complication (%)	5-Year Survival	10-Year Survival
Bacle [13]	87	MCT, CTA, Revision, Post-trauma, POA	12.5	29%	-	93%
Beck [42]	29	CTA, FS, Revision	8.5	14%	-	93%
Cuff [30]	76	MCT, CTA	5.1	-	94%	-
Ek [14]	46	MCT, CTA < 65 years	7.8	38%	98%	88%
Favard [15]	489	MCT, CTA, POA	4.5	18%	-	89%
Guery [16]	60	MCT, CTA, Revision, Post-trauma, RA	5.8	15%	-	91%
Gallinet [17]	422	Acute fracture	2.3	13%	-	91%

MCT: Massive Cuff Tear, CTA: Cuff Tear Arthropathy, FS: Fracture Sequelae, POA: Primary OsteoArthritis, RA: Rheumatoid Arthritis, ANHH: Avascular Necrosis of Humeral Head.

## 5. Conclusions

The ten-year revision-free survival of a primary RSA for cuff-deficient shoulders, primary osteoarthritis, and rheumatoid arthritis is above 90%; however, some indications are associated with a higher complication rate and a lower survivorship (tumor, fracture sequelae, and revision RSA). Younger patients are also more likely to have postoperative complications and revision surgeries. The most frequent complications are instability, infection, and humerus-related complications.

The survival of an RSA depends on the diagnosis and on the patient’s age and gender. Surgeons should be aware of the high rates of complications and the lower survival rates of RSAs in younger patients, in males, and in RSAs for revision surgery. While the 10-year survival rate free of revision is over 95% in RSAs for MCTs and failed cuff repairs, it drops down to 80% or below in RSAs for fracture sequelae, tumors, and previous failed arthroplasties. The two main complications are low-grade infections and humeral loosening because of proximal humeral bone loss.

## Figures and Tables

**Table 1:** Diagnosis for reverse shoulder arthroplasty.

Diagnosis	Frequency
Cuff Tear Arthropathy (Hamada IV and V)	497 (30.9%)
Revision of a Failed Arthroplasty, including:	239 (14.8%)
Revision of Hemi-Arthroplasty	138 (8.5%)
Revision of Anatomic Shoulder Arrthroplasty	85 (5.2%)
Revision of Reverse Shoulder Arrthroplasty	16 (1%)
Massive Cuff Tear (Hamada I, II, and III)	188 (11.7%)
Proximal Humerus Fracture Sequelae	185 (11.5%)
Failed Cuff Repair	153 (9.5%)
Primary Osteoarthritis	142 (8.9%)
Acute Proximal Humerus Fracture	84 (5.2%)
Rheumatoid Arthritis	53 (3.3%)
Malignant Tumor	25 (1.6%)
Instability Arthropathy	22 (1.4%)
Other (septic arthritis, osteonecrosis)	24 (1.5%)

**Table 2:** Most frequent postoperative complications and their surgical treatments.

Complication	Frequency	Mean Delay (mo.)	Surgical Treatment
Infection	61 (3.8%)	26.3 (±28.4) (0.6–112)	Washout (21) Explantation (12) Two-stage exchange (10) One-stage exchange (8) Washout + Mobile implant change (4) Glenoid component revision (2) Revision to HA (1)
Instability	45 (2.8%)	19.2 (±32.7) (0–139)	Open reduction + Mobile implant change (17) Closed reduction (16) Open reduction without implant change (5) Humeral component revision (2) Glenoid component revision (2) One-stage RSA exchange (2) Revision to HA (1)
Glenoid loosening	25 (1.6%)	45.5 (±52.3) (0.4–154)	Revision to HA (9) Glenoid component revision (7) One-stage RSA exchange (5) Explantation (1)
Humeral loosening	23 (1.4%)	62.8 (±55.1) (9–180)	Humeral component revision (9) One-stage RSA exchange (5)
Humeral fracture	23 (1.4%)	49.5 (±38.1) (0.1–121)	Open reduction and internal fixation (11) Humeral component revision (3)
Nerve injury	22 (1.4%)	-	-
Scapular spine fracture	11 (0.7%)	47.4 (±66.4) (1–167)	Open reduction and internal fixation (2)
Acromial fracture	6 (0.4%)	2.1 (±1.3) (1–4)	-
Severe stiffness	4 (0.3%)	-	Arthroscopic arthrolysis (3) Humeral component revision (1)

**Table 3:** Reoperation-free and revision-free or reintervention-free survivals in primary and revision RSA.

	Primary RSA n = 1282	Revision RSA for Failed Arthroplasty n = 329	*p*-Value for the Difference between Primary and Revision RSA
Reoperation-free survival (%)			
5-year	97.3 (96–98)	93.2 (89–96)	0.002 *
10-year	95.3 (93–97)	89.9 (84–94)	
15-year	94.1 (91–96)	89.9 (84–94)	
Revision-free survival (%)			
5-year	95.8 (94–97)	84.3 (78–89)	<0.001 *
10-year	91.0 (88–93)	80.4 (74–86)	
15-year	85.3 (79–90)	71.5 (58–81)	

* log-rank test, *p* < 0.05; RSA: Reverse Shoulder Arthroplasty.

**Table 4:** Complications and revision surgeries according to gender and age.

	N =	Complication Rate (%)	Reoperation Rate (%)	Revision Rate (%)	5-Year Revision-Free Survival	10-Year Revision Free Survival
Gender						
Male	402	23.1	6.7	10.4	90.3	83.2
Female	1207	14.2	3.0	5.6	95.4	91.5
*p*-Value		<0.001 *	<0.001 *	<0.001 *	<0.001 *	<0.001 *
Age at RSA						
<60 years	118	34.7	8.5	19.5	86.7	75.7
60–69 years	327	20.5	3.7	9.5	93.3	88.8
70–79 years	830	14.5	3.9	5.4	95.0	91.3
≥80 years	331	11.5	3.0	3.3	95.9	94.3
*p*-Value		<0.001 *	0.068	<0.001 *	<0.001 *	<0.001 *

* *p* < 0.05.

**Table 5:** Revision rate according to age stratified by gender.

Revision Rate (%)	<60 Years (n = 118)	60–69 Years (n = 327)	70–79 Years (n = 830)	≥80 Years (n = 331)	*p*-Value According to Age
All RSA (n = 1611)					
Women (n = 1207)	14.3	9.0	4.5	2.9	<0.001 *
Men (n = 404)	26.5	10.4	8.5	3.5	<0.001 *
*p*-Value (according to gender)	0.096	0.701	0.034 *	0.817	
Revision RSA (n = 238)					
Women (n = 168)	20.0	19.6	5.5	17.4	0.078
Men (n = 70)	36.4	18.2	18.2	0	0.261
*p*-Value (according to gender)	0.314	1	0.087	1	

* *p* < 0.05.

**Table 6:** Complications and revision surgeries according to the indication for RSA.

	Complication Rate (%)	Reoperation Rate (%)	Revision Rate (%)	5-Year Revision-Free Survival	10-Year Revision Free Survival
Revision RSA	32.6	8.0	16.0	84.8	80.9
Revision of HA	32.8	7.3	13.9	86.8	83.2
Revision of ATSA	25	8.3	11.9	87.8	83.3
Revision of RSA	66.7	11.1	55.6	50.0	50.0
CTA (Hamada IV and V)	12.3	2.8	5.0	95.7	91.9
MCT (Hamada I, II, and III)	17.6	5.9	3.7	96.4	95.3
Fracture Sequelae	20.0	3.8	10.8	92.9	83.9
Failed Cuff Repair	11.8	3.9	3.3	96.1	96.1
Primary Osteoarthritis	8.5	0.7	3.5	97.5	90.3
Acute Fracture	6.0	1.2	2.4	98.7	88.9
Rheumatoid Arthritis	9.4	0.0	1.9	97.6	97.6
Tumor	56	12.0	20.0	86.2	59.1
Instability Arthropathy	4.5	4.5	0.0	100.0	100.0

ATSA: Anatomic Total Shoulder Arthroplasty, CTA: Cuff Tear Arthropathy, HA: Hemi-arthroplasty, MCT: Massive Cuff Tear, RSA: Reverse Shoulder Arthroplasty.

## Data Availability

The data presented in this study are available from the corresponding author upon request. The data are not publicly available due to ethical restrictions.
